# Cdk5 links with DNA damage response and cancer

**DOI:** 10.1186/s12943-017-0611-1

**Published:** 2017-03-14

**Authors:** Wan Liu, Jun Li, Yu-Shu Song, Yue Li, Yu-Hong Jia, Hai-Dong Zhao

**Affiliations:** 1 Department of Breast Surgery, The Second Affiliated Hospital of Dalian Medical University, Zhongshan Road 467, Dalian, 116023 China; 2 Department of Pathophysiology, Dalian Medical University, Lvshun South Road West 9, Dalian, 116044 China

**Keywords:** Cyclin dependent kinase 5, DNA damage response, Apoptosis, Cancer, Targeted cancer therapy

## Abstract

As an atypical member of cyclin dependent kinase family, Cyclin dependent kinase 5 (Cdk5) is considered as a neuron-specific kinase in the past decade due to the abundant existence of its activator p35 in post-mitotic neurons. Recent studies show that Cdk5 participates in a series of biological and pathological processes in non-neuronal cells, and is generally dysregulated in various cancer cells. The inhibition or knockdown of Cdk5 has been proven to play an anti-cancer role through various mechanisms, and can synergize the killing effect of chemotherapeutics. DNA damage response (DDR) is a series of regulatory events including DNA damage, cell-cycle arrest, regulation of DNA replication, and repair or bypass of DNA damage to ensure the maintenance of genomic stability and cell viability. Here we describe the regulatory mechanisms of Cdk5, its controversial roles in apoptosis and focus on its links to DDR and cancer.

## Background

Cyclin dependent kinases (Cdks) are a large family of serine/threonine kinases, consisting of 20 Cdks (Cdk1-Cdk20) and 5 Cdk-like (Cdkl) proteins (Cdkl1-Cdkl5), numbered by their discoveries [1]. Cyclin dependent kinase 5 (Cdk5) is a member of Cdk family which share a basic structure and high conserved sequence, with the catalytic site that binds to ATP sandwiched between N- and C-terminal lobes [2]. Unlike other Cdks, Cdk5 generally neither participates in the regulatory progression of cell cycle control nor is activated by cyclins, but is activated by binding with non-cyclin Cdk5 activators Cdk5R1 (p35) and Cdk5R2 (p39), or their respective truncations p25 and p29, all of which are originally found abundant in post-mitotic neurons. The predominant distribution of Cdk5 activators in neurons makes Cdk5 a neuron-specific Cdk [3].

Over the last decade Cdk5 has been proven to play a critical role in essential neuronal functions, including control of cytoskeletal architecture and dynamics, axonal guidance, neuronal migration, cell adhesion, etc.; and participate in the pathological changes in neurodegenerative diseases [4]. However, there is growing evidence of its roles outside the nervous system. Cdk5 has been found to participate in several biological processes of extraneuronal activities, such as gene expression, cell migration, apoptosis, myogenesis, etc.; and pathological processes including cancer, senescence, diabetes, immune dysfunction and inflammation [5, 6]. Cdk5 is generally dysregulated in various types of cancer and is linked with cancerous characteristics and prognoses [6], making it a novel biomarker and promising therapeutic target in cancer treatment.

Recent studies have also shown that the disruption of DNA damage response (DDR) plays an important role in cancer development and treatment [7]. The basic function of DDR is to repair the DNA damage and keep the integrity and stability of genome, which is related to tumorigenesis and progression. DDR includes the event of DNA repair process itself and other events such as the detection of DNA lesions, cell-cycle arrest, etc. [8, 9]. Pearl and his colleges have compiled a data set of 450 genes encoding proteins that are integral to the DDR [10], revealing their roles and therapeutic potentials in cancer and treatment. Cdk5 takes part in DDR process mainly by phosphorylating some of the critical DDR proteins such as ataxia–telangiectasia mutated (ATM) and apurinic/apyrimidinic endonuclease 1 (Ape1), and the inhibition of this kinase activity has been proven to regulate DDR process and cancer progression [11, 12]. Here we focus on the regulation of Cdk5, its dual roles in apoptosis, and its links with DDR and cancer development and treatment. To our best known, this would be the first review that elaborates the roles of Cdk5 in DDR and its links with cancer characteristics.

## Regulation of Cdk5

**Fig. 1:**
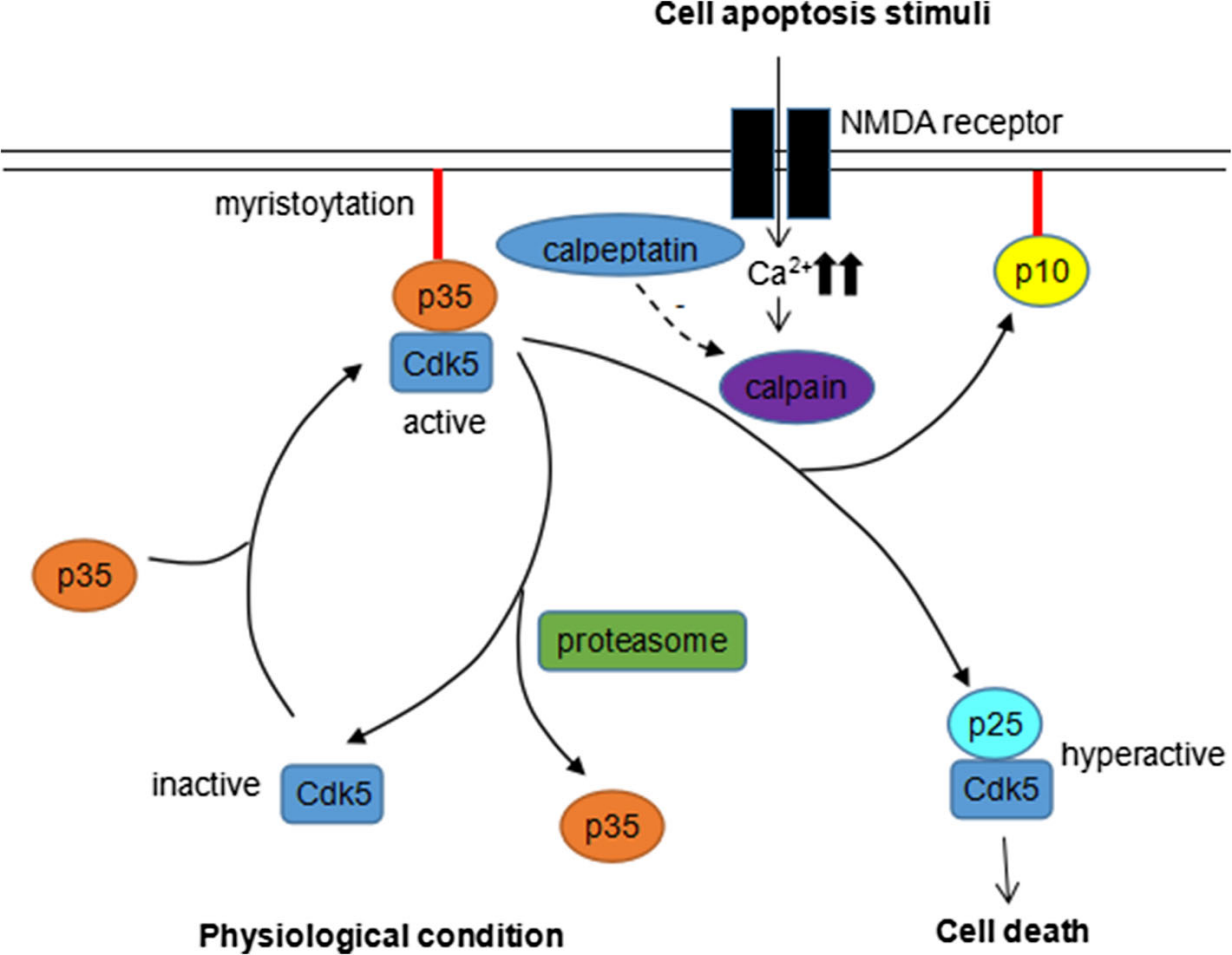
The regulatory mechanism of Cdk5 in neurons. Cdk5 itself does not own an enzymatic activity. Physiologically Cdk5 is activated by binding with p35 and the complex of Cdk5/p35 is concomitantly recruited to membrane via the myristoylation of p35. p35 is a short-lived protein which is soon degraded by proteasome, resulting in transient activation of Cdk5. When cell faces cell death signals, the NMDAR on cellular membrane is activated and membrane permeability to calcium is increased, leading to activation of calpain protein. Activated Calpain cleaves p35 into p25 fragment, a much more stable protein than p35, resulting in translocation of Cdk5/p25 complex into nucleus and prolonged activation of Cdk5, which induces pathological signaling pathways of cell death

The regulatory mechanism of Cdk5 is well elucidated in neurons. Cdk5 itself does not own an enzymatic activity. Physiologically Cdk5 is activated by binding with p35, and the complex of Cdk5/p35 is predominantly cytoplasmic and membrane-associated due to myristoylation of p35 [13]. p35 is a short-lived protein with a life span of 20-30mins, which can be phosphorylated by activated Cdk5 [14], leading to its ubiquitination and degradation by proteasome [15]. Its degradation by proteasome results in an attenuated activity of Cdk5 and forms a negative-feedback modulation of Cdk5 activity [16–18]. When cell faces cell death signals, the N-methyl-D-aspartate receptor (NMDAR) on cellular membrane is activated and membrane permeability to calcium is increased, which subsequently activates calpain protein. Calpain owns a proteolytic activity which cleaves p35 into p25 fragment, a much more stable protein than p35. The cleavage results in a translocation of Cdk5/p25 complex into nucleus and prolonged activation of Cdk5, which induces pathological signaling pathways of cell death [19, 20] [Fig. 1]. Both calpastatin and calpeptin, an endogenous and an exogenous calpain-inhibitor respectively, can attenuate Cdk5 activity by suppressing the cleavage of p35 into p25 [21, 22]. Similarly, p39 can also be cleaved into a more stable subunit p29, which results in Cdk5 hyperactivation and relocation [23]. Yet the function and significance of p29 remain to be characterized.

**Fig. 2:**
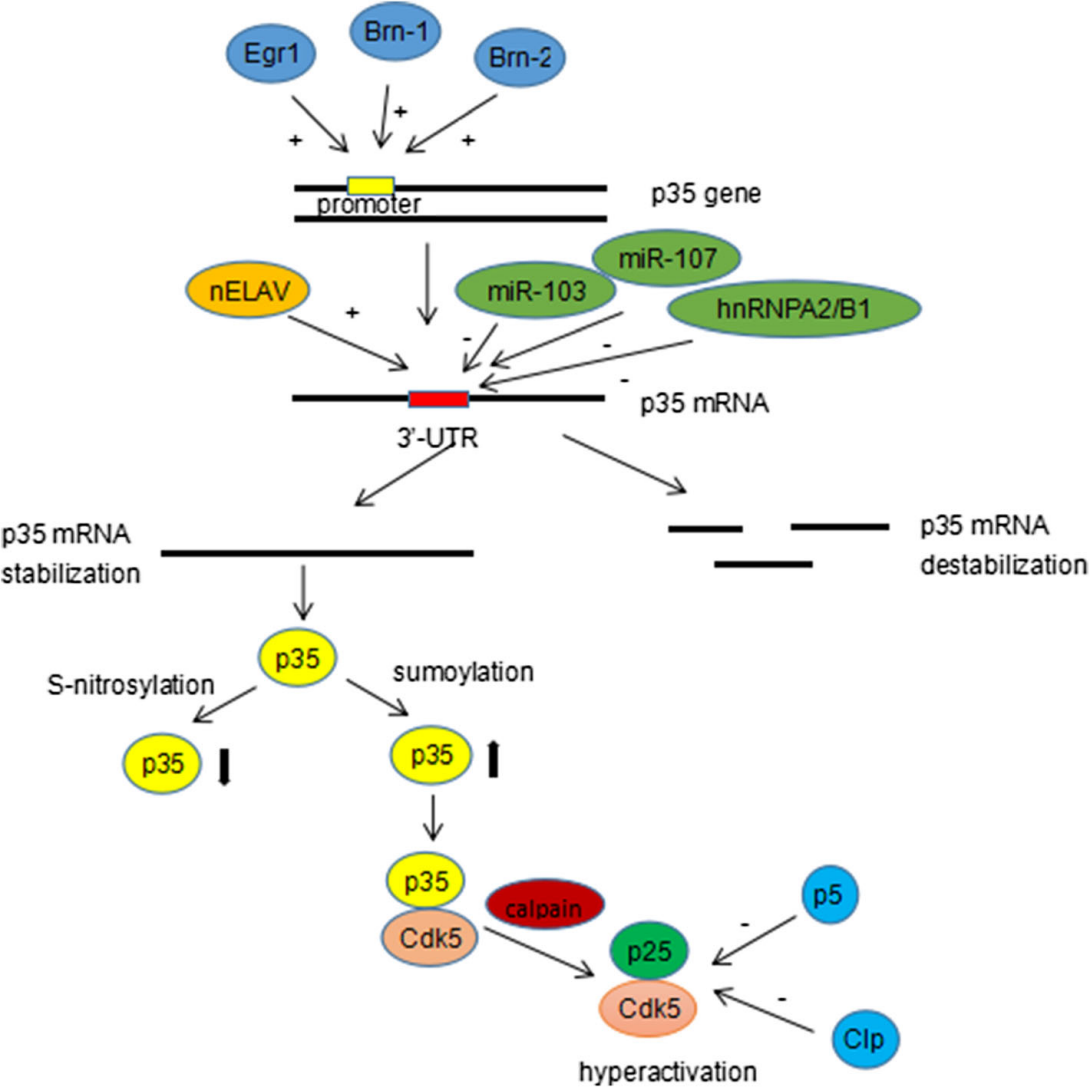
The activation of Cdk5 is regulated by modulation of p35 in different levels. In transcriptional level, the transcription factors Egr1, Brn-1 and Brn-2 can bind to the promoter of p35 gene and positively regulate its mRNA expression. After transcription, the binding of nELAV to p35 mRNA 3’-UTR can positively control its mRNA stability, while hnRNPA2/B1, miR-103 and miR-107 have a negative effect resulting in mRNA destabilization. Post-translational protein modifications of p35, sumoylation and S-nitrosylation, can respectively increase and restrain Cdk5 activity. The over-expression of two small peptides derived as truncations of p35, CIp and p5, can selectively inhibit Cdk5/p25 hyper-activation without affecting endogenous Cdk5/p35 activity

Since Cdk5 plays its enzymatic activity through its active form Cdk-p35/Cdk-p25 complex, the activation of Cdk5 is mainly regulated by the regulation of Cdk5 activator p35/p25. It has been reported that the binding of transcription factors such as early growth response gene 1 (Egr1), POU-homeodomain transcription factors Pou3f3 (Brn1) and Pou3f2 (Brn2) to the promoter of p35 gene can positively regulate p35 mRNA expression and Mitogen-activated protein kinase (MAPK) signaling dependent up-regulation of p35 in neuronal and non-neuronal cells, resulting in enhanced enzyme activity of Cdk5 [24, 25]. Cdk5 kinase activity can also be regulated through post transcriptional modulation of p35 mRNA. The long 3’ untranslated region (UTR) of p35 mRNA, which contains putative GY box motif and AU-rich elements, has been reported to affect mRNA stability in neuronal and non-neuronal cells [26]. The binding of neuron-specific embryonic lethal abnormal vision (nELAV) to p35 mRNA 3’-UTR can positively control p35 mRNA stability, while heterogeneous nuclear ribonucleoproteins A2/B1 (hnRNPA2/B1), miR-103 and miR-107 miRNAs are reported to have a negative effect on p35 mRNA stability [27, 28]. Besides, post-translational modifications of p35 protein, such as sumoylation and S-nitrosylation, can respectively increase and restrain Cdk5 activity [29, 30]. Furthermore, the over-expression of two small peptides derived as truncations of p35, Cdk5 inhibitory peptide (CIp) and peptide 5 (p5), can selectively inhibit hyperactivation of Cdk5/p25 without affecting endogenous Cdk5/p35 activity [31, 32] [Fig. 2].

Apart from the canonical Cdk5 activators p35 and p39, recent studies have identified more factors that can regulate Cdk5 activity. Cdk5 activity can be restricted by its interaction with cyclin E, which can further influence synaptic plasticity and memory formation in neurons [33]. Cyclin D1 over-expression results in attenuated Cdk5/p35 activity, probably by its competition with p35 for Cdk5 in cortical neuronal cell [34]. Cyclin G1 is reported to activate Cdk5 in lung and colorectal cancer cells by protein interaction [35]. Glutathione-S-transferase pi 1 (GSTP1) is demonstrated to inhibit Cdk5 activity directly by binding to Cdk5 and dislodging p25/p35, and indirectly by suppressing oxidative stress-induced Cdk5 hyperactivation in neuronal and cancer cells [36]. Nestin, an intermediate filament protein, can bind with Cdk5/p35 complex and create sequestration which depresses p35 processing, resulting in attenuated Cdk5-p25 activation and subsequent restriction of myoblast differentiation [37].

## Cdk5’s dual roles in apoptosis

Studies have shown that Cdk5 plays a protective role in cell survival. In INS 832/13 and primary β-cells, the knockdown of Cdk5 or p35 leads to a significant decrease in focal adhesion kinase (Fak) activity and its downstream phosphorylation of Akt at ser473, which is known as a protective factor of cell survival; and an obvious increase in caspase-3 cleavages, which is a symbol of apoptosis [38]. The anti-apoptosis role of Cdk5 has also been proved in thyroid cancer cells [39] and prostate cancer cells [40].

However, there is also enough evidence revealing its roles of pro-apoptosis in various cells. In neurons, Cdk5 activation can mediate an aberrant S-phase entry and death of cells by different signaling pathways, including inhibition of Cdh1-mediated activation of anaphase-promoting complex/cyclosome (APC/C) [41]; phosphorylation of C-terminus of Hsc70-interacting protein (CHIP) [42], myocyte enhancer factor 2D (MEF2D) [43], and NMDAR subunit 2A (NR2A) [44]; and remodeling of chromatin structure [45]. In prostate cancer cells exposed to retinoic acid (RA) treatment (a traditional anti-cancer drug), Cdk5 is overactivated and cell proliferation is suppressed. Both the inhibition and knockdown of Cdk5 can obstruct RA-triggered apoptosis and caspase 3 activation [22]. Cdk5 is also proved to be an apoptosis-enhancing signal in ischemic injury. Cdk5 mediates ischemia-induced cell death through phosphorylation and inactivation of peroxiredoxin 2 (Prx2) and MEF2D, an antioxidant enzyme and an survival promoting transcription factor respectively. Moreover, the treatment with Cdk5 inhibitors can suppress the ischemia-induced cell death in renal tubular cells, suggesting the potential value of Cdk5 for ischemia-relating diseases and renal preservation [46, 47].

## Involvement of Cdk5 in DDR

Cells are challenged by tens of thousands of lesions on their DNA everyday, which vary from creating DNA crosslinks to block DNA replication to causing DNA double-strand breaks (DSBs) to damage genome integrity. If unrepaired or aberrantly repaired, these DNA damages can be accumulated and commit cells to apoptosis or induce aberrant cellular signaling pathways leading to cancer [48]. Thus cells have developed a sophisticated net of regulatory pathways collectively termed DNA damage response. The principle of DDR is to ensure the lesions of DNA damage are well-repaired before the genetic materials are inherited to progeny. The events contain the detection of DNA damage, cell-cycle arrest, regulation of DNA replication, and repair or bypass of DNA damage to ensure the maintenance of genomic stability and cell viability [49]. DDR has been elucidated to be linked with tumorigenesis and treatment and drug-resistance of tumor [50]. Here we focus on the roles of Cdk5 in DDR through different pathways.

As a widely proline directed serine/threonine protein kinase, Cdk5 is not directly involved in DNA repair, but regulates DDR by phosphorylating various key regulators of DDR process. At least 450 proteins integral to DDR have been analyzed, and one of these DDR proteins is Ape1, which is essential in the base excision repair (BER) pathway, one of the key repair mechanisms for DNA damage [50]. Ape1 can cleave apurinic/apyrimidinic (AP) site caused by DNA damage, and facilitate the following repair processes of BER by subsequent enzymes [51]. Huang and his collegues demonstrated that in neurons Cdk5 is hyperactivated and Ape1 is phosphorylated at Thr 232 upon oxidative stress and DNA damage [11]. The phosphorylation of Ape1 leads to significantly attenuated AP endonuclease activity and failure of DNA damage repair. Inhibition of Cdk5 can dephosphorylate Ape1 and rescue the AP endonuclease activity of Ape1 and contribute to DNA damage repair [11], suggesting that the hyperactivation of Cdk5 promotes DNA damage process through phosphorylation of Ape1 and subsequent inactivation of AP endonuclease activity, and the inhibition of Cdk5 facilitates DNA damage repair.

**Fig. 3:**
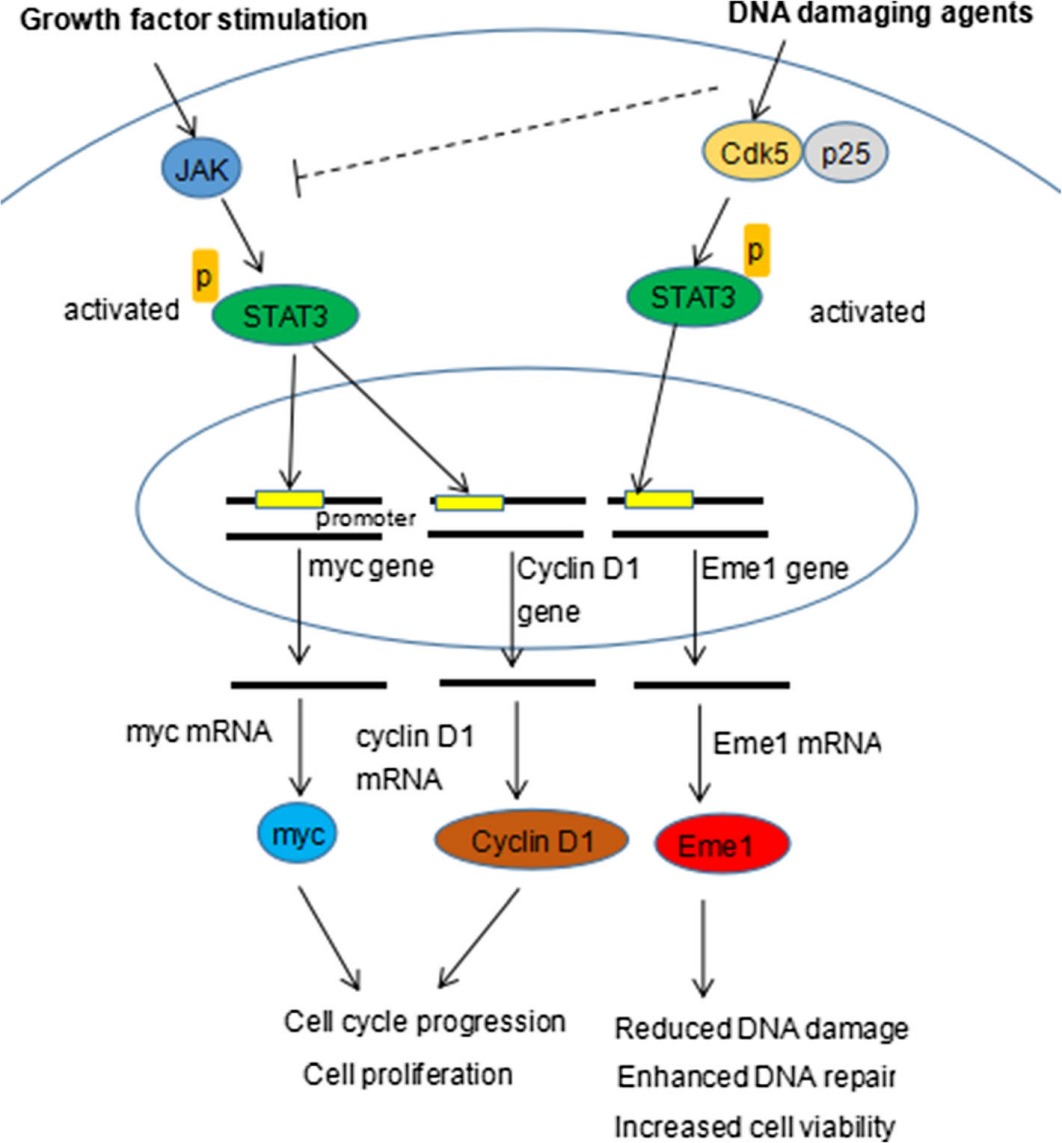
Cdk5 takes part in DDR through STAT3 pathway. In growing cancer cells, the constitutive activation of STAT3 via JAK-mediated phosphorylation on tyrosine 705 allows the nuclear translocation and DNA binding of STAT3 and up-regulation of cyclin D1 gene and myc gene, leading to cell cycle activation and proliferation. When cells are treated with topoisomerase I inhibitor (a DNA damaging agent), the activation of STAT3 by JAK is inhibited, while the expression and activity of Cdk5 are increased in response to DNA damaging agent. Up-regulated Cdk5 associates with STAT3 and phosphorylates it on serine 727, leading to activation of STAT3. The activated STAT3 then translocates to nucleus and interacts with the promoter of Eme1 gene, promoting the rescue of broken replication forks caused by DNA damage

Cdk5 also influence the DDR process by regulating the signal transducer and activator of transcription 3 (STAT3)-mediated up-regulation of essential meiotic structure-specific endonuclease 1 (Eme1), an endonuclease that is implicated in rescuing broken replication forks in response to DNA damaging agents [52, 53]. STAT3 proteins are cytoplasmic transcription factors that translocate to the nucleus and induce cell cycle progression in response to growth factor stimulation [54]. In growing cancer cells, the constitutive activation of STAT3 via JAK-mediated phosphorylation on tyrosine 705 allows the nuclear translocation and DNA binding of STAT3 and up-regulation of several genes such as cyclin D1 gene and myc gene, two kinds of proliferative genes, leading to cell cycle activation and proliferation. When cells are treated with topoisomerase I inhibitor (a DNA damaging agent), the activation of STAT3 by JAK is inhibited, leading to decreased expression of cyclin D1 and myc. However, both the expression and activity of Cdk5 are increased in response to DNA damaging agent, and co-immunoprecipitation and pulldown experiments show that up-regulated Cdk5 associates with STAT3 and promotes phosphorylation of STAT3 on serine 727 and subsequent activation of STAT3. The activated STAT3 then translocates to nucleus and interacts with the promoter of Eme1 gene, promoting the rescue of broken replication forks caused by DNA damage. The inhibition of Cdk5 can inhibit the STAT3-mediated up-regulation of Eme1 and result in impaired DNA repair and increased DNA damage and further decreased cell viability [52] [Fig. 3]. Taken all together, the up-regulation of Cdk5 is a result of DNA damage stimulation and in turn induces DNA repair through STAT3-mediated up-expression of Eme1, which may confer cancer cells resistance to chemotherapy treatment.

The roles of Cdk5 in DNA damage repair have also been reported to be mediated by interaction with ATM kinase, a crucial regulator in DDR process [55]. ATM is demonstrated to favor homologous recombination (HR) after being recruited to DSBs by MRE11-RAD50-NBS1 (MRN) complex [56]. ATM is involved in presynapsis of HR by recruiting and activating nuclease enzymes including BRCA1, CtIP, EXO1 and BLM, which resect DSB ends to generate 3’-ssDNA overhangs and hence facilitate RAD51 nucleofilament formation [57, 58]. Besides, recent study shows ATM is additionally engaged in the later steps of HR after DSB end resection and RAD51 loading [59]. Cdk5 is proved to be responsible for ATM activation by promoting the phosphorylation of ATM at Ser 794 and subsequent autophosphorylation, and mediate DNA damage signaling, cell cycle checkpoint and neuronal death in post-mitotic neurons [12]. There are also reports that Cdk5 play a role in DDR by phosphorylating Prx2 (an antioxidative enzyme) [60], P53 (a tumor suppressor protein) [61] and p19INK4d (a member of cell cycle inhibitor family INK4) [62], whereas the signaling pathways need further exploration and elucidation.

## Cdk5 links with cancer

Tumorigenesis is a series of complex processes, with a variety of aberrant signaling changes which confer cells cancerous characteristics. Surgery therapy is no more the only way for cancer treatment, looking for the potential biomarkers and effective target therapies has become the current hotspot [63]. The emerging knowledge of the roles of Cdk5 in cancer indicates that it is a potential biomarker for diagnosis and prognosis prediction for cancer. Cdk5 is reported to be over-expressed or hyperactivated in various cancer tissues and tumor cell lines. In patients with lung cancer it is associated with clinical pathological characteristics and poorer prognoses [64]. In head and neck squamous cell carcinoma, aberrant over-expression of Cdk5 significantly induces tumor cell motility and epithelial mesenchymal transition (EMT), which is considered as a pivotal process of cancer metastasis [65]. In hepatocelluar carcinoma (HCC), over-expression and hyperactivation of Cdk5 play an oncogenic activity by inducing proliferation and clonogenic growth of HCC [66]. In pancreatic cancer cells, Cdk5 is widely active. Blockade of Cdk5 can remarkably downregulate the two active forms of Ral proteins RalA-GTP and RalB-GTP, and Rho-GTP and Rac-GTP levels, all of which play an important role in oncogenic Ras-induced neoplasia, tumor progression and metastasis, revealing the important roles of Cdk5 in cancer progression [67]. The roles of Cdk5 in pancreatic cancer formation and progression via Ras-Ral signaling have been further proven by rescue test, in which constitutively activating RalA-GTP and RalB-GTP in pancreatic cancer cells expressing dominant-negative Cdk5 significantly rescued the effects of Cdk5 inhibition [67]. In prostate cancer cells, Cdk5 promotes cell growth in an androgen receptor (AR)- independent way and maintains high AKT kinase activity, which is known to correlate with prostate cancer progression and considered as a critical growth-promoting pathway in prostate cancer cells [68]. There are also reports that Cdk5 mediates prostate cancer progression through STAT3 and AR signaling [69]. In breast cancer cells treated with transforming growth factor beta 1 (TGF-β1, an EMT inducer), both p35 and Cdk5 are up-regulated and hyperactivated, leading to subsequent EMT and cancer cell motility by phosphorylating Fak, which is known to be involved in cellular adhesion and spreading processes [70]. Our work also shows Cdk5 and p35 are significantly increased in clinical breast cancer tissues and in breast cancer cell lines exposed to paclitaxel at transcriptional and translational levels.

Though Cdk5 is generally up-regulated in most types of cancer, it has also been reported down-regulated in certain types of cancer. For example, in human gastric cancer tissues, the mRNA level and protein level of Cdk5 and p35 are both significantly reduced, and the down-regulation is linked with poorer prognoses [71]. Cellular experiments show a lack of Cdk5 in nucleus in different gastric cancer cell lines, but presence of Cdk5 in both the nucleus and cytoplasm in normal gastric epithelial cell line. NS-0011, which inhibits the translocation of Cdk5 from nucleus to cytoplasm, can suppress the proliferation and xenograft tumorigenesis of gastric cancer cells [71]. The effects of disrupting the localization of Cdk5 by drugs reveal a novel mechanism to limit its pathological signalings in cancer cells [71].

Taken all together, Cdk5 participates in tumorigenesis, progression and metastasis of different cancers through various signaling pathways, and its inhibition and knockdown have been proven to be effective in restraining cancer cell progression. All the studies provide potential targets of Cdk5-relating pathways for cancer treatment.

## Implication of Cdk5 in cancer treatment

Cancer is currently the most threatening challenge worldwide and the development of novel targeted cancer therapy (TCT) is of utmost clinical importance. Different from the traditional cellular toxic chemo-therapy, TCT plays its stronger anti-cancer effect by targeting specifically to the aberrant characteristics of cancer cells and meanwhile reduces the toxic effects on normal cells [72]. Up to now a total of 30 TCT drugs have been approved by America Food and Drug administration (FDA) since 1960s, targeting at epidermal growth factor receptor (EGFR), hairy-related 2 (HER-2), AR signaling, Poly-(ADP-ribose) polymerase (PARP), mammalian target of rapamycin (mTOR) signaling, etc. No specific drugs that target Cdk5 signaling have been approved, nevertheless a wave of emerging studies reveal that Cdk5 is a promising therapeutic target in cancer treatment. Dinaciclib (formerly SCH727965), a potent and selective small molecule inhibitor of Cdk2, Cdk5, Cdk1 and Cdk9, is the first Cdks inhibitor to enter the clinic trail. Its promising anti-cancer results and acceptable safety profile have been respectively proven in preclinical studies and human phase I trial. In preclinical model of ovarian cancer, SCH727965 has been shown to synergize with cisplatin in killing cancer cells [73]. In phase I trial of SCH727965, subjects with advanced malignancies experienced acceptable and tolerable dose-limiting toxicities, including orthostatic hypotension, elevated uric acid, nausea, anemia, neutropenia, etc. [74]. Besides, As Cdk5 is hyperactivated in a degradation-dependent pathway by proteasome calpain, we speculate that the proteasome inhibitor Bortezomib (Velcade), which has been approved by FDA for mantle cell lymphoma treatment [75], may play anti-cancer function to some extent by regulating Cdk5-related pathways in other cancers.

Cdk5 also plays an important role in treatment of chemo-resistant cancers. For example, in cervical cancer the expression of cyclin I is up-regulated by cisplatin treatment, which in turn confers cancer cells resistance to cisplatin by activating Cdk5 and its anti-apoptosis effect. Knockdown of Cdk5 with siRNA can significantly increase the sensitivity to cisplatin in Hela cell lines with over-expressed cyclin I [76]. Similar results are also observed in Cdk5-inhibited HCC cells [66] and Cdk5-depleted ovarian cancer cell lines [77], in which cancer cells exhibit higher sensitivity to DNA damaging agents.

The inhibition of Cdk5 can also reduce the side effects of some chemo-therapy strategies. For example, Paclitaxel is one of the widely used chemotherapeutics for various solid tumors, while its continuous dose is reported to induce tumor dissemination and metastasis. Both specific inhibition and knockdown of Cdk5 are able to suppress tumor migrational and invasive ability by impairing the paclitaxel-induced invadopodia formation and EMT process [78, 79]. Taken all together, Cdk5-related signalings are promising targets for cancer treatment, and the combination of Cdk5 inhibition and chemo-therapy can synergize the killing effect and reduce side effects, providing a potential strategy for better clinical application of chemotherapeutic agents.

## Conclusion

The level of Cdk5 and its kinase activity are generally dysregulated in various cancer tissues and tumor cell lines, and the dysregulation is linked with DDR, apoptosis, tumorigenesis, EMT, metastasis and other cancerous characteristics via a series of complex mechanisms. Cdk5 is considered as a potential biomarker in different cancers and a promising target for cancer treatment. The inhibition of Cdk5 can effectively suppress cancer proliferation and metastasis in cancer cells, and can synergize the killing effect of chemotherapeutics and meanwhile reduce its side effects, providing a better strategy for the clinical application of chemotherapeutics. However, the knowledge of the biological and pathological functions of Cdk5 in DDR and cancer is still limited, more efforts need to be taken to explore and exploit this kinase better.

## Abbreviations

AP: Apurinic/apyrimidinic
APC/C: Anaphase-promoting complex/cyclosome
Ape1: Apurinic/apyrimidinic endonuclease 1
AR: Androgen receptor
ATM: Ataxia–telangiectasia mutated
BER: Base excision repair
Cdk5: Cyclin dependent kinase 5
Cdkl: Cdk-like
Cdks: Cyclin dependent kinases
CHIP: C-terminus of Hsc70-interacting protein
CIp: Cdk5 inhibitory peptide
DDR: DNA damage response
DSBs: DNA double-strand breaks
EGFR: Epidermal growth factor receptor
Egr1: Early growth response gene 1
Eme1: Essential meiotic structure-specific endonuclease 1
EMT: Epithelial mesenchymal transition
Fak: Focal adhesion kinase
FDA: America food and drug administration
GSTP1: Glutathione-S-transferase pi 1
HCC: Hepatocelluar carcinoma
HER-2: Hairy-related 2
hnRNPA2/B1: Heterogeneous nuclear ribonucleoproteins A2/B1
HR: Homologous recombination
MAPK: Mitogen-activated protein kinase
MEF2D: Myocyte enhancer factor 2D
MRN: MRE11-RAD50-NBS1
mTOR: Mammalian target of rapamycin
nELAV: Neuron-specific embryonic lethal abnormal vision
NMDAR: N-methyl-D-aspartate receptor
NR2A: NMDAR subunit 2A
p5: Peptide 5
PARP: Poly-(ADP-ribose) polymerase
Prx2: Peroxiredoxin 2
RA: Retinoic acid
STAT3: Signal transducer and activator of transcription 3
TCT: Targeted cancer therapy
TGF-β1: Transforming growth factor beta 1
UTR: Untranslated region.
